# Integrated Analysis of Seed microRNA and mRNA Transcriptome Reveals Important Functional Genes and microRNA-Targets in the Process of Walnut (*Juglans regia*) Seed Oil Accumulation

**DOI:** 10.3390/ijms21239093

**Published:** 2020-11-29

**Authors:** Xinchi Zhao, Guiyan Yang, Xiaoqiang Liu, Zhongdong Yu, Shaobing Peng

**Affiliations:** 1 Laboratory of Walnut Research Center, College of Forestry, Northwest A & F University, Yangling 712100, Shaanxi, China; zhaoxinchi@nwsuaf.edu.cn (X.Z.); yangguiyan@nwsuaf.edu.cn (G.Y.); yuzhongdong001@nwsuaf.edu.cn (Z.Y.); 2 Department of Foreign Languages, Northwest A & F University, Yangling 712100, Shaanxi, China; jacava@163.com

**Keywords:** *Juglans regia*, oil accumulation, microRNA, regulatory network

## Abstract

Walnut (*Juglans regia*) is known as a promising woody oil crop with abundant polyunsaturated fatty acids in its kernel. However, the regulation mechanism of walnut oil accumulation and fatty acid metabolism is still poorly understood, which restricted the breeding and genetic improvement of high-quality oil-bearing walnuts. To reveal the molecular mechanism of walnut oil accumulation, considering the potential regulation of microRNA (miRNA) in seed development, in this study, the oil content of walnut kernel on the 80th, 100th and 120th day after flowering (DAF) was tested and the corresponding proportions are 11.51%, 40.40% and 53.20%. Between DAF of 80th~120th, the content of stearic acid and oleic acid tended to increase, but the proportion of other fatty acids tended to decrease. Meanwhile, comparative transcriptome and sRNA-seq analysis on three stages (80th, 100th and 120th DAF), found 204 conserved miRNAs and 554 novel miRNAs in walnut kernels, among which 104 key genes related to walnut oil accumulation were screened. The phospholipid:diacylglycerol acyltransferase metabolic pathway may contribute more to oil accumulation in walnut. 16 miRNA-mRNA regulatory modules related to walnut oil accumulation and fatty acid synthesis were constructed. 8 known miRNAs and 9 novel miRNAs regulate 28 genes involved in fatty acid (FA) metabolism and lipid synthesis. Among them, jre-miRn105, jre-miRn434, jre-miR477d and jre-miR156a.2 are key miRNAs that regulate walnut FA synthesis. Jre-miRn411 and jre-miR399a.1 are closely related to oil accumulation. These data provide new insights and lay the foundation for subsequent studies on walnut FA synthesis and oil accumulation.

## 1. Introduction

*Juglans regia*, a kind of woody oil plant, is commonly known as Persian walnut or English walnut. Walnut oil is rich in tocopherol, squalene, oleic acid, linoleic acid, linolenic acid and sterols [1]. As a kind of functional oil, walnut oil has a good health care effect on human body and multiple economic values, leading to a growing demand for walnut oil [2,3]. Therefore, it is of great value and significance to clarify the mechanism of walnut seed oil anabolic process, which lays a foundation for the breeding of high-yield oil-type walnuts and genetic improvement of walnut, and provides an important theoretical basis.

Oils mainly exist in the form of fatty acid glycerides, generally triacylglycerols (TAG) in plants seeds, which are stored in oil bodies as energy and biosynthetic precursors [4]. Existing studies have fully confirmed that the process of lipid accumulation has a complex regulatory relationship at the transcriptional level. LEAFY COTYLEDON1 (LEC1) can induce the expression of fusca3 (*FUS3*), abscisic acid insenseitive3 (*ABI3*) and leafy cotyledon2 (*LEC2*) [5]. Overexpression of *LEC1* up-regulated more than half of the known enzyme-encoding genes in plastid FA synthesis pathway [6]. LEC2 can affect the synthesis of FA and TAG by regulating fatty acid desaturase (*FAD*) *8*, *FAD7*, *FAD3*, diacylglycerol O-acyltransferase (*DGAT*), glycerol-3-phosphate acyltransferases (*GPAT*), lysophosphatidyl acyltransferase (*LPAT*) and phosphatidic acid phosphatase (*PAP*) [7]. Moreover, LEC2 is considered to be a key upstream transcription factor (TF) directly controlled wrinkled1 (*WRI1*) expression [8]. WRI1 is an important TF in plant oil synthesis pathway, overexpression of *WRI1* can increase plant oil content [9]. ABI4 and ABI5 play an important role in regulating diacylglycerol O-acyltransferase 1 (*DGAT1*) expression, which is an important rate-limiting enzyme in TAG biosynthesis [10,11,12]. Although these genes have been studied in soybeans, rapeseed and *Arabidopsis*, they have not been involved in woody oil crops, especially walnuts.

MicroRNAs (miRNAs) are endogenous, single stranded small non-coding RNAs. Previous studies have shown that miRNAs play an important role in a series of biochemical reactions, such as plant growth, development and stress resistance [13,14,15]. The process of oil accumulation in plants relies on the expression of a variety of lipid biosynthesis genes, which mainly occur at the transcriptional level, as well as complex transcriptional and post-transcriptional control mechanisms [16]. Cao et al. [17] identified 13 differentially expressed miRNAs between ordinary and high-oleic acid mutant safflower seeds. Li et al. [18] has described the miRNA-transcription factor regulatory network in seed development of sea buckthorn and hypothesized that 12 possible regulatory modules play a combinatorial regulatory role in seed development and oil accumulation. The artificial miRNA amiR159b constructed based on the miR159b gene of *Arabidopsis thaliana* successfully changed the fatty acid composition of *Arabidopsis* seed oil [19].

Previous study has found that “Xiangling” (a cultivar of *J. regia*) oil accumulation increased most rapidly 70th~100th day after flowering (DAF), and then slowed down. The oil conversion rate was highest on the 80th DAF [20]. Oil bodies began to appear in the cotyledons of walnut on 80th DAF, and oil bodies and protein bodies filled cotyledons on 120th DAF [21]. Recently, it has been reported that some miRNAs have been found in walnut female flower buds and leaf buds using high-throughput sequencing technology, which may be related to the regulation of walnut flower bud formation and female flower differentiation [22]. However, until now, little is known about miRNAs in the lipid biosynthesis process of walnut. To systematically understand the regulatory network between miRNAs and mRNAs related to walnut oil biosynthesis, miRNAs and mRNA sequences during the accumulation period of walnut oil were analyzed. The high-throughput sequencing and bioinformatics tools were used in our research to identify conserved and novel miRNAs as well as their potential targets. In the current study, we found some miRNA-mRNA regulatory modules which are potentially related to walnut seed kernel oil accumulation. Our results may provide new insights into the regulatory mechanisms of miRNAs during walnut seed kernel oil accumulation.

## 2. Results

### 2.1. Dynamic Analysis of Oil Content and Fatty Acid

The moisture content and oil content of the samples in each period was calculated (Table S1). Moisture content of walnut kernels decreased continuously during the three sampled stages as 74.10%, 39.80% and 25.97%, respectively. Meanwhile, the oil content shows an upward trend rising rapidly from 11.51% to 40.40% and then to 53.20%. The FAs composition of kernels in three stages of walnut fruit development were measured (Table 1). A total of 16 FAs was detected in these three periods, including 7 kinds of saturated FAs and 9 kinds of unsaturated FAs. The top five maximum FAs components in kernels were palmitic acid (C16:0), stearic acid (C18:0), oleic acid (C18:1), linoleic acid (C18:2, n-6), and linolenic acid (C18:3, n-3). The total relative contents of these five FAs were all above 97% in G1 (80th DAF), G2 (100th DAF), and G3 (120th DAF). It was worth noting that C18:0 and C18:1 generally showed an increasing trend during the three sampled stages, while the rest FAs showed a dynamic equilibrium or decreasing trend. Principal component analysis more intuitively shows the difference is in FAs composition of oils in the three periods. G1, G2 and G3 are clearly separated in the PC1 × PC2 score plot (Figure S1).

### 2.2. Key Genes in Accumulate Period of Walnut Oil

To study the important genes in the accumulation stage of walnut oil, and nine cDNA libraries that included three stages were constructed. A total of 60.36 Gb of raw data were obtained from Illumina sequencing, and 37,154,970, 43,644,082, 40,643,444, 48,26,5040, 44,838,030, 37,342,352, 43,313,330, 41,040,198, and 40,294,380 clean reads were obtained after removing the low-quality reads and adapter sequences. The proportion of clean reads mapped to the walnut genome in each library was above 96%, and the proportion of uniquely mapped reads ranged from 82.24% to 88.46% (Table S2). Principal component analysis was performed to compare the transcriptome characteristics of nine samples intuitively (Figure S2). The samples at different stages were significantly separated, while the replicates were closely spaced, indicating that a large number of genes were altered in expression levels at these three stages.

**Figure 1:**
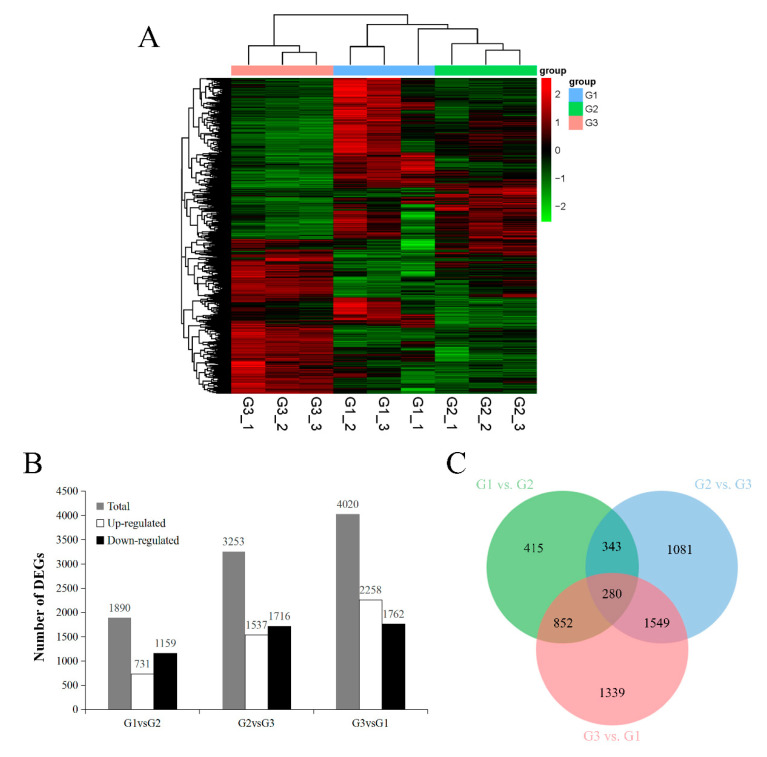
Analysis of differential gene expression in walnut seed kernel at different developmental stages. (**A**) Heatmap and clustering analysis of expression levels of DEGs. (**B**) Up-regulated and down-regulated the amount of DEGs. (**C**) The Venn diagram of DEGs.

Furthermore, the gene expression profiles of different samples at three stages were compared. The changes of RNA-seq at different stages were detected by clustering analysis of differentially expressed genes (DEGs) expression pattern (Figure 1A). DEGs showed a significant time-specific expression pattern. Besides, we found 1890 DEGs between G1 and G2 (731 genes were up-regulated and 1159 genes were down-regulated), 3253 DEGs between G2 and G3 (1537 were up-regulated and 1716 were down-regulated), 4020 DEGs between G3 and G1 (2258 were up-regulated and 1762 were down-regulated) (Figure 1B).

**Figure 2:**
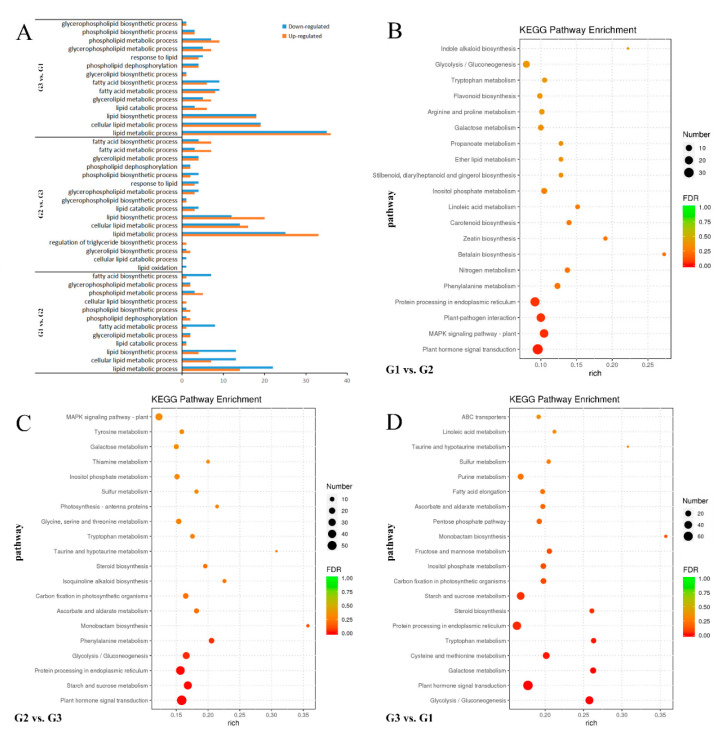
Enrichment analysis of all DEGs. (**A**) DEGs related to FA synthesis screened. (**B**–**D**) The top 20 KEGG pathways with the most significant enrichment of G1 vs. G2, G2 vs. G3, G3 vs. G1, accordingly.

To better understand the function of these DEGs in walnut oil synthesis, Gene Ontology (GO) and Kyoto Encyclopedia of Genes and Genomes (KEGG) annotation analyses were performed 18 GO terms related to the metabolism and synthesis of walnut oil were selected from the Biological process (Figure 2A). There are 104, 116 and 118 pathways were categorized from a pairwise comparison in the enrichment analysis of KEGG pathway between G2 vs. G1, G3 vs. G2, and G3 vs. G1, respectively (Table S3). The top 20 enriched KEGG pathways of each comparison are shown in Figure 2B–D. Among the DEGs of G2 vs. G1 and G3 vs. G2, the most significantly enriched KEGG pathway was “Plant hormone signal transduction” (ko04075), followed by “Protein processing in endoplasmic reticulum” (ko04141) and “Phenylalanine metabolism” (ko00360). “Starch and sucrose metabolism” (ko00500) and “Glycolysis/gluconeogenesis” (ko00010) were also significantly enriched in G3 vs. G2 DEGs. Among the DEGs of G3 vs. G1, the most significantly enriched KEGG pathway was “Glycolysis/gluconeogenesis” (ko00010), followed by “Plant hormone signal transduction” (ko04075), “Galactose metabolism” (ko00052), “Cysteine and methionine metabolism” (ko00270) and “Tryptophan metabolism” (ko00380). There are more down-regulated genes in these pathways than up-regulated genes. Finally, 8 major pathways related to walnut oil synthesis were selected from 13 “Lipid metabolism pathways”, including “Linoleic acid metabolism” (ko00591), “Fatty acid biosynthesis” (ko00061), “Fatty acid degradation” (ko00071), “Biosynthesis of unsaturated fatty acids” (ko01040), “Arachidonic acid metabolism” (ko00590), “α-Linolenic acid metabolism” (ko00592), “Fatty acid elongation” (ko00062), “Glycerolipid metabolism” (ko00561). After KEGG and GO enrichment analysis, it was identified that 104 DEGs may be importantly related to the limiting rate of oil synthesis in the late stage of walnut seed maturity (Table S4).

**Figure 3:**
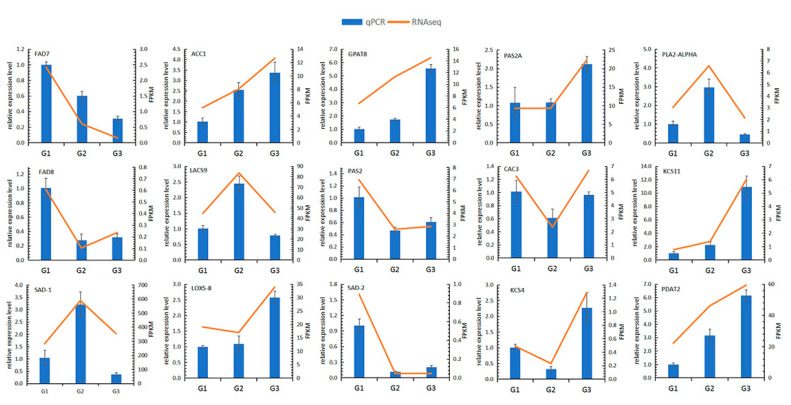
QRT-PCR verifies the quality of transcriptome sequencing. The blue bar graph represents the relative expression level of the sample (left *y*-axis), and the yellow line graph represents the normalized result (FPKM) of the sample in the transcriptome (right *y*-axis).

To verify the accuracy of sequencing results, 15 genes were selected to verify if their trends were consistent with the sequencing results. The qRT-PCR results showed that the relative expression levels of selected genes were consistent with the transcriptome sequencing results (Figure 3), and the Pearson correlation coefficients was 0.8659, confirming that the transcriptome sequencing results were highly reliable (Figure S3A).

### 2.3. Sequence Characteristics of Small RNA in J. regia

**Figure 4:**
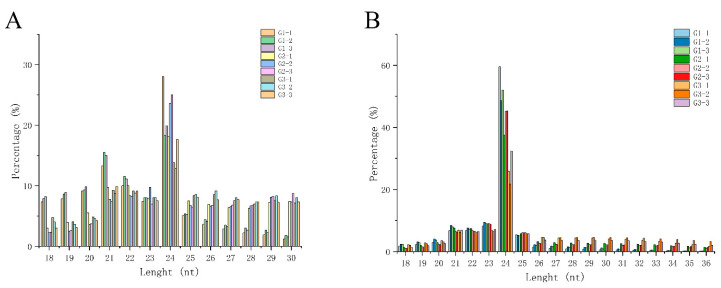
The length distribution of small RNA in the walnut kernel at different developmental stages. (**A**) Length distribution of clean reads. (**B**) Length distribution of unique reads.

In nine small RNA libraries, high-throughput sequencing produced clean reads (≥18nt) ranging from 15.95 to 30.19 million. The length of 24 nt is the most abundant class among clean and unique reads (Figure 4A,B). Ribosomal RNA (rRNA) accounts for the majority of non-coding RNA in total and unique reads. The detailed information about these 9 libraries was listed in Table S5 provides. A large number of unique reads nonmatched to the Rfam database will be used for subsequent analysis.

### 2.4. Known miRNA and Novel Candidate miRNAs in J. regia Kernels

**Figure 5:**
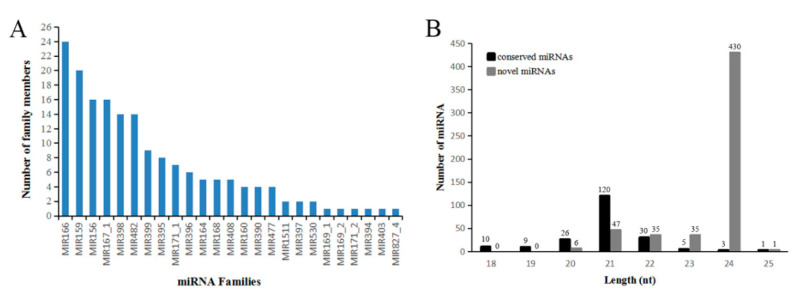
miRNAs identified from walnut kernels. (**A**) Families and members distribution of known miRNA. (**B**) Length distribution of known miRNA and novel miRNA.

A total of 204 known miRNAs were identified in nine libraries. These conserved miRNAs were grouped into 25 families, of which MIR166 was the largest family with 24 members, followed by MIR159 family with 20 members, the MIR156 and MIR167_1 with 16 members each. Among the remaining 21 families, 6 families contained only one member, and 15 families contained 2~14 members (Figure 5A, Table S6).

The sequences nonmatched to Rfam and miRBase database were compared with the genome to predict the novel miRNAs by Mireap. 554 novel candidate miRNAs were predicted. Some novel miRNAs with the same mature sequence but different precursors were considered to belong to a novel miRNA family. These novel miRNA candidates were named in the form of “jre-miRn plus number”, using letters to distinguish members from the same novel miRNA family, and “-3p” and “-5p” to distinguish mature sequences produced on different arms of the same precursor (e.g., jre-miRn88-3p, jre-miRn88-5p). The length of the new miRNA precursors ranged from 64 to 103 nt; the minimum free energy (MFE) ranged from 18 to 80.8 kcal/mol; and the minimum free energy index (MFEI) ranged from 0.85 to 2.1 (Table S7). Most of the novel miRNAs expression levels were quite low in consistent with the results obtained from many experiments [23,24].

The length distribution of the miRNAs ranged from 18 to 25 nt (Figure 5B). Most of the mature sequences in conservative miRNAs were concentrated in 21 nt, while most of the novel candidate miRNAs were concentrated in 24 nt. This miRNA distribution was very similar to that reported in camphor trees [25].

### 2.5. Differential Expression of miRNAs and Functional Analysis

**Figure 6:**
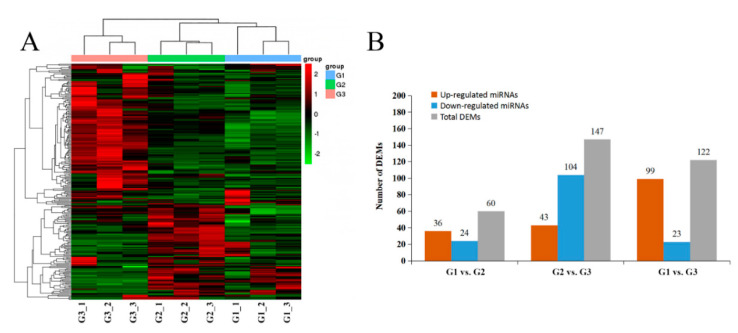
The expression profile of DEMs at different developmental stages of kernels. (**A**) Heatmap and clustering analysis of DEMs. (**B**) The number and characteristics of DEMs among different groups.

Most miRNAs showed significant differential expression (Figure 6A) and 232 DEMs were defined at three stages (Figure 6B). Sixty DEMs were identified in G1 vs. G2 (24 down-regulated, 36 up-regulated), one hundred and forty-six DEMs (104 down-regulated, 42 up-regulated) were identified in G2 vs. G3, and one hundred and twenty-two DEMs (23 down-regulated, 99 up-regulated) were identified in G1 vs. G3.

To better understand the function of these miRNAs, psRobot_tar was used to predict the target genes of DEMs. A total of 1784 target genes were predicted. These genes were targeted by 174 miRNAs with an average of 10.25 target genes regulated by each miRNA (Table S8). Interestingly, out of 102 novel miRNA, 44 contained only one target gene, while jre-miRn105, jre-miRn213, and jre-miRn434 contained more than 100 target genes. Furthermore, three members of the miR156 family: jre-miR156a, jre-miR156c, and jre-miR156e, most of their target genes, belonged to the SBP (SQUAMOSA promoter-binding protein) TF family members.

The GO enrichment analysis summarizes the functions of target genes into three major categories, namely biological process (BP), molecular function (MF) and cell composition (CC), which are subdivided into 1565 terms in these three categories (Table S9). In the BP category, the most significantly enriched terms were “Phenylpropanoid metabolic process” (GO: 0009698), “Lignin metabolic process” (GO: 0009808), and “Phenylpropanoid catabolic process” (GO: 0046271). In the category of MF, the most significantly enriched terms were mainly related to “Hydroquinone: oxygen oxidoreductase activity” (GO:0052716), “DNA binding” (GO:0003677), and “Oxidoreductase activity, acting on diphenols and related substances as donors, oxygen as acceptor” (GO:0016682). In the CC category, the most significantly enriched terms were grouped into “Nucleus” (GO:00056340), “Membrane-bounded organelle” (GO:0043227), and “Intracellular membrane-bounded organelle” (GO:0043231). These target genes were separated into 5 categories, 18 subcategories, and 88 pathways totally in KEGG pathway annotation. The number of pathways related to “Metabolism” is the highest among the 5 categories (Table S10), of which the most is “Carbohydrate metabolism” (ko00640, ko00030, ko00620, ko00052, ko00630, ko00562, ko00650, ko00053, ko00010, ko00051, ko00520, ko00020, ko00500, ko00040), followed by “lipid metabolism” (ko00561, ko00061, ko00062, ko00100, ko01040, ko00072, ko00565, ko00590, ko00564, ko00071, ko00600, ko00073, ko00592).

**Figure 7:**
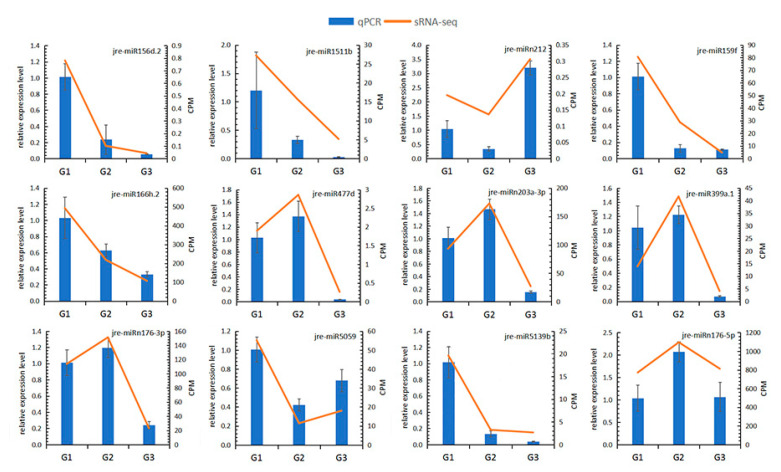
QRT-PCR verified the consistency of the relative expression levels of 15 random miRNAs with the small RNA sequencing results. The blue bar graph represents the relative expression level of the sample (left *y*-axis), and the yellow line graph represents the normalized result (CPM) of the sample in sRNA-seq (right *y*-axis).

Twelve miRNAs were randomly selected for qRT-PCR verification to verify the consistency of the miRNA and sRNA sequencing results. The relative expression levels of 12 miRNAs were consistent with the results of small RNA sequencing (Figure 7). The significant Pearson correlation coefficient r = 0.8077 indicates that the data obtained by sRNA-Seq were reliable (Figure S3B).

### 2.6. Involvement of miRNAs in Walnut Oil Synthesis Pathways

**Figure 8:**
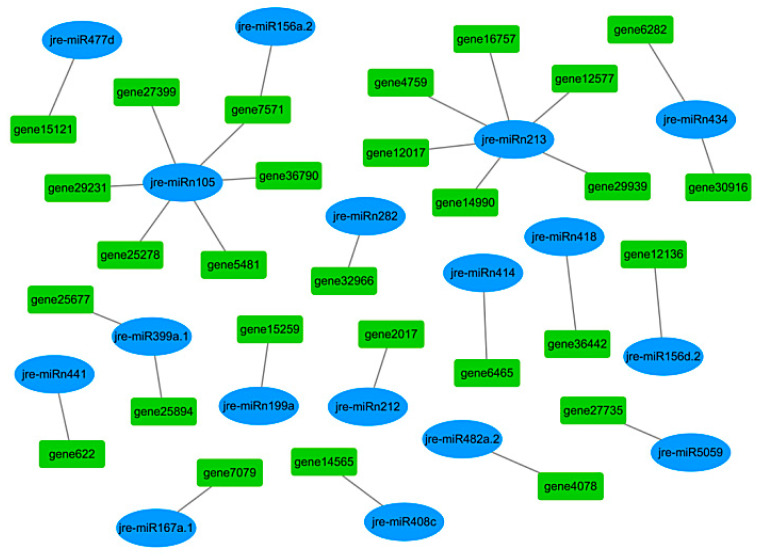
Potential miRNA-mRNA regulatory module is related to oil accumulation during walnut fruit development.

Among 1784 target genes, 28 related to walnut oil biosynthesis were screened out, corresponding to 17 DEMs. A miRNA-target gene regulatory network was constructed to explore the relationship between miRNA and its targets (Figure 8). Sixteen independent regulatory modules were related to walnut oil accumulation. Thirteen miRNAs target a single gene, and four miRNAs target multiple genes that were related to walnut oil biosynthesis.

Some of the target genes encode the same enzymes and are genes with the same functions. Among them, palmitoyl-acyl carrier protein thioesterase (*FATB*, *gene7571* and *gene36442*) was targeted by jre-miR156a.2, jre-miRn105, and jre-miRn418. Phospholipase D delta (*PLDδ*, *gene25677* and *gene25894*) was targeted by jre-miR399a.1. Delta 8-fatty-acid desaturase 2 (*SLD2*, *gene12136* and *gene6282*), targeted by jre-miR156d.2 and jre-miRn434, play a major role in the process of ceramide biosynthesis [26]. Four miRNAs correspondingly regulate multiple target genes related to lipid synthesis. 3-ketoacyl-CoA synthase 11 (*KCS11*, *gene5481*), very-long-chain (3R)-3-hydroxyacyl-CoA dehydratase PASTICCINO 2A (*PAS2A*, *gene25278*), beta-carotene hydroxylase 2 (*CA2*, *gene27399*), putative 3,4-dihydroxy-2-butanone kinase (*DHBK*, *gene36790*), and UTP--glucose-1-phosphate uridylyltransferase 3 (*UGP2*, *gene29231*) were targeted by jre-miRn105. Protein ABHD17C (*gene12577*), protein ABHD17B (*gene16757*), aldehyde dehydrogenase family 7 member A1 (*gene12017*), diphosphomevalonate decarboxylase MVD2 (*gene29939*), monogalactosyldiacylglycerol synthase (*gene4759*), and beta-galactosidase (*gene14990*) were targeted by jre-miRn213. Moreover, 3-oxoacyl-[acyl-carrier-protein] reductase 4 (*FabG*, *gene30916*) was targeted by jre-miRn434. Acetyl-CoA carboxylase 1 (*ACC1*, *gene15121*) was targeted by jre-miR477d. Other genes, including acyl-CoA thioesterase 2 (*ACOT2*, *gene7079*), acetyl-CoA acetyltransferase 1 (*AAT1*, *gene14565*), type IV inositol polyphosphate 5-phosphatase 9 (*IP5P9*, gene4078), phospholipase A I (*PLA1*, *gene27735*), putative quinone-oxidoreductase homolog (*gene15259*), stearoyl-[acyl-carrier-protein] 9-desaturase (*SAD*, *gene2017*), 3-ketoacyl-CoA synthase 6 (*KCS6*, gene32966), 4-coumarate--CoA ligase-like 5 (*4CLL5*, *gene6465*), and probable 1-acyl-sn-glycerol-3-phosphate acyltransferase 4 (*LPAT4*, *gene622*), were targeted by jre-miR167a.1, jre-miR408c, jre-miR482a.2, jre-miR5059, jre-miRn199a, jre-miRn212, and jre-miRn441, respectively.

## 3. Discussion

*J. regia* is an important woody oil plant with high economic value. In recent years, high-throughput sequencing and bioinformatics tools were used to confirmed that some miRNAs were related to lipid metabolism in some oilseed crops, such as *Glycine max* [27], *Brassica napus* [28], *Carya cathayensis* [29] and *Elaeis guineensis* [30]. However, miRNA-mRNA regulatory mechanism shows species specificity. Clarifying the biological mechanism of fatty acid synthesis and oil accumulation is one of the effective ways to increase walnut oil production.

According to the previous studies on the dynamic accumulation of walnut oil [20], three developmental stages of walnut oil accumulation from fast to slow (80th, 100th and 120th DAF) were selected. The proportion of PUFAs in walnut oil significantly decreased from 80th DAF to 120th DAF. Instead, monounsaturated fatty acids (MUFA) significantly increased (Table 1). This phenomenon of fatty acid changes is similar to that observed in safflower [31].

In order to explore the molecular mechanism of walnut oil accumulation rate-limiting and FAs composition change. In current study, nine cDNA libraries for transcriptome sequencing at three stages were constructed, and over than 96% of clean reads in each library were mapped to the walnut reference genome, 5859 genes were significantly differentially expressed (Figure 1C). What is more, the changes of γ-linolenic acid (C18:3, n-6) and α-linolenic acid (C18:3, n-3) are positively correlated with the expression changes of *FAD7* and *FAD8*. Considering that fatty acid desaturases and acyl-carrier-protein desaturases are key enzymes for unsaturated FAs synthesis, for instance, FAD3, FAD7 and FAD8 catalyze the desaturation reaction of linoleic acid to make it become linolenic acid [32,33]. It is easy to think that *FAD7* and *FAD8* may contribute to walnut linolenic acid biosynthesis, which was consistent with previous study that *FAD7* is a key gene for the accumulation of linolenic acid in walnut kernels [34].

**Figure 9:**
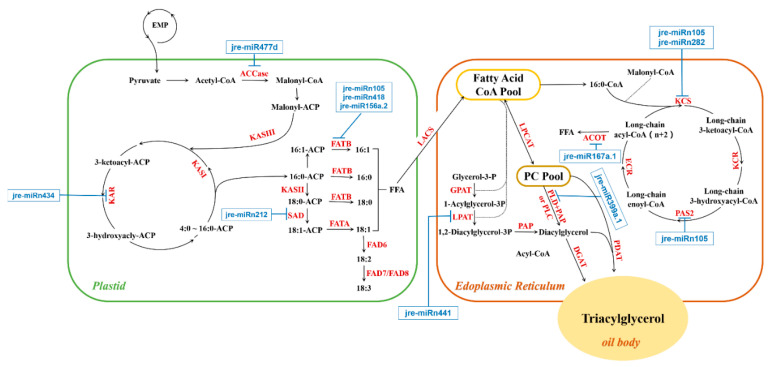
Overview of miRNAs and genes regulation patterns in FA and TAG synthesis pathways. The red letters are genes, and the blue letters are miRNAs.

In addition, we found that *gene29135* and *gene3132* encode the SAD, but both genes show opposite trends between G1 and G2. However, oleic acid content in walnut oil was significantly increased between G1 and G2. Since SAD can convert stearoyl-ACP to oleoyl-ACP by introducing a cis double bond on the acyl chain of stearoyl-ACP [35], thereby promoting the accumulation of oleic acid. The current results suggested that *gene29135* and *gene3132* may be key genes in walnut oleic acid synthesis. Furthermore, there were significant differences in the expression of 3-ketoacyl-CoA synthase (*KCS*), very long-chain 3-oxoacyl-CoA reductase (*KAR*), long chain acyl-CoA synthetase (*LACS*) and very long-chain (3R)-3-hydroxyacyl-CoA dehydratase 2 (*PAS2*) (Table S4, Figure 9), indicating the synthesis and dynamic changes of very long-chain FAs during the maturation of walnut seeds are closely related to these genes [36].

At present, there are two main pathways for TAG synthesis have been reported in higher plants. One is from diacylglycerols (DAG) and acyl-CoA under the catalysis of DGAT [37,38], and the other is by transferring the fatty acyl group of phosphatidylcholine (PC) to DAG through phospholipid:diacylglycerol acyltransferase (PDAT) [39]. DGAT and PDAT are important rate-limiting enzymes in TAG synthesis and necessary for fruit development [40,41]. *DGAT3* is highly expressed in all three stages (FPKM > 100). While, in this study, only *PDAT1-1*, *PDAT1-2* and *PDAT2* showed significant differential expression, suggesting that PDAT metabolic pathway may have more influence on the accumulation of walnut oil. The significant differential expression of *LEC2*, *ABI4* and *ABI5* indicates that these three TFs may have important contributions in the process of walnut oil accumulation. Interestingly, *LEC1* was not detected in walnut transcripts, so TF regulation of walnut lipid accumulation may be different from *Arabidopsis* [26].

Plant unique SPL TFs were regulated by miR156 [42,43], which was similar as the results we obtained. MiR156 family member jre-miR156a.2 and jre-miR156d.2 also regulate *FATB* and *SLD2*. FATB is closely related to the accumulation of palmitic acid in seeds [44], and *SLD2* is related to the biosynthesis of ceramide [45]. In this study, we identified that jre-miR477d targets to *ACC1*. As a multifunctional enzyme that catalyzes the carboxylation of acetyl-CoA, ACC1 is used in the synthesis of FAs in plastids and in various biosynthetic pathways including FAs extension in the cytoplasm [46,47]. These results imply that jre-miR477d may relate to the synthesis of long-chain FAs and the improvement of the efficiency of oil biosynthesis in walnut (Figure 9). Similarly, miR477 in *Camellia oleifera* was considered to be a key miRNA regulating oil accumulation because it regulates the target ACC carboxytransferase subunit alpha (*CAC3*) [48]. Besides, the jre-miR5139b targets two squalene epoxidase 3 (*SQE3*, *gene32107* and *gene24076*) to regulate the synthesis of squalene in walnut seeds. Some novel miRNAs, such as jre-miRn105 targeting *KCS11*, *FATB* and *PAS2A*, jre-miRn212 targeting *SAD*, jre-miRn282 targeting *KCS6*, jre-miRn418 targeting *FATB*, jre-miRn434 targeting *FabG* and jre-miRn441 targeting *LPAT4*, almost all of those target genes are rate-limiting genes in unsaturated FAs and TAG synthesis (Figure 9). Above results and reports suggested that these novel miRNAs may also play an important role in oil accumulate or FAs biosynthesis.

In conclusion, as an important factor in post-transcriptional regulation, miRNAs in walnut are rarely reported at present, and the role of miRNA in walnut oil biosynthesis has not been reported yet. This research shows for the first time the miRNA regulatory network that may exist in the biosynthesis of walnut oil. Our work also screened out some key genes during the accumulation period of walnut oil and analyzed their expression patterns. We hope that the walnut oil biosynthesis mechanism can be further improved on this basis. The results of this study provide valuable information for the biosynthesis and improvement of walnut oil, also provide a new idea for molecular breeding of walnut for oilseeds.

## 4. Materials and Methods

### 4.1. Plant Materials

“Xiangling” walnut (a cultivar of *J. regia*) was used. All the samples were collected from Weihe River Experimental Station of Northwest A&F University (34.20° N, 108.40° E). The total annual average solar radiation of the station is 109.68 kcal/cm^2^, the annual average temperature is 13.3 °C, and the annual average precipitation is 715 mm. We randomly selected nine eight-year-old healthy and disease-free “Xiangling” walnut trees with the same growth level. Forty-five mixed fruit obtained from three trees were regarded as a treatment (total three independent biological replicates). The fruit was sampled every 20 days from the 80th DAF and marked as G1 (80th DAF), G2 (100th DAF), G3 (120th DAF). The fruit was removed the green husks quickly. Then walnut kernels were fetched out, packed into the self-sealing bags, and transported to the laboratory. All samples were flash-frozen in liquid nitrogen and stored in the refrigerator at −80 °C until use.

### 4.2. Analysis of Oil Content and Fatty Acid Composition

The walnut kernels (50.00 g, each) were dried to constant weight at 105 °C and grounded to a homogenized powder. 2.00 g of powder in a water-free and oil-free filter paper bag was used to extract crude fat in a Soxhlet apparatus at 60 °C for 12 h using petroleum ether (boiling range 30~60 °C) as the extractant. The methylated crude fat was analyzed by gas chromatography-mass spectrometry (GC-MS) equipped with hydrogen flame ionization detector and HP-FFAP chromatographic column (Agilent Technologies, Santa Clara, CA, USA). Heptadecanoic acid methyl ester (C17:0) was used as an internal standard. Working conditions of GC: inlet temperature was 260 °C, split ratio was 10:1, detector temperature was 280 °C. Initial column temperature was 210 °C, hold 8 min, then temperature was increased by 20 °C/min to 230 °C and maintained at this temperature for 6 min.

### 4.3. Total RNA Isolation, cDNA Library Construction and Transcriptome Sequencing

The total RNA was extracted by HiPure HP Plant RNA Mini Kit (Magen, Guangzhou, China) according to the manufacturer’s instructions. The purity and concentrations of RNAs were determined by 1% agar-gel electrophoresis and NanoDrop NC 2000 (Thermo Scientific, Wilmington, DE, USA). RNA integrity was assessed using RNA Nano 6000 Kit of Agilent Bioanalyzer 2100 system (Agilent Technologies, Santa Clara, CA, USA), mRNA was purified by the interaction of the poly (A) tails and magnetic oligo (dT) beads. The first strand of cDNA was synthesized by 6 bp random primers and reverse transcriptase with RNA as template. Then, the second strand of cDNA was synthesized with the first strand of cDNA as template. Finally, nine RNA-seq libraries (three stages with three independent biological replicates) were double-ended sequenced using next-generation sequencing based on Illumina platform at Shanghai Personal Biotechnology Cp., Ltd. (Shanghai, China).

### 4.4. Small RNA Library Construction and Small RNA Sequencing

NEBNext Multiplex Small RNA library Prep Kit (New England Biolabs, Inc., Hitchin, UK) was used for small RNA libraries construction according to the manufacturer’s instructions. 10 μg of total RNA from 9 independent samples was ligated with 3′ adapter and 5′ adapter with the T4 RNA ligase. RNA was reverse-transcribed to synthesize double-stranded cDNA using SuperscriptII reverse transcriptase. DNA fragments were enriched by PCR. Then, products were separated using PAGE gel by fragment size. Fragment size and distribution of DNA library were verified for quality control of PCR enriched fragments using Agilent 2100. The total library concentration was detected using Picogreen. These libraries were single-end sequenced on the HiSeq 2500 platform at Shanghai Personal Biotechnology Cp., Ltd. (Shanghai, China).

### 4.5. Processing and Analysis of Transcriptome

The raw data were obtained by sequencing the cDNA library. Subsequently, the adaptor sequences, reads with a quality score < Q20, reads with a final length < 25 bp, and reads with uncertain bases, were removed from raw data. The clean reads were mapped to the *J. regia* reference genome [49] using the HISAT2 [50] software.

DEGs analysis was performed using DESeq v.1.18.0. [51]. The selection of DEGs was based on |log2 fold change| > 1 with *p*-value < 0.05. The GO enrichment analysis was performed using topGO: annotated DEGs were used to calculate the gene list and number for each term. The *p*-value was calculated to find out the GO term by hypergeometric distribution method (the criterion for significant enrichment was *p*-value < 0.05). Compared with the whole genome background, significant enrichment of differential genes was used to determine the main biological functions of differential gene exercise. Furthermore, pathway annotation of DEGs was performed using KEGG database (http://www.genome.jp/kegg/).

### 4.6. Bioinformatics Analysis of Small RNA Sequences

The clean reads were applied for small RNA analysis. The number of clean reads was counted whose sequence length is more than 18 nt and less than 36 nt. The identical sequence in a single sample was deduplicated and the sequence abundance was counted to obtain unique reads for subsequent analysis. The unique reads were compared with the Rfam (13.0) database using BLAST. Four types of known non-coding RNAs (rRNA, tRNA, snRNA, and snoRNA) were screened whose screening criterion is no more than two mismatches. Unique reads, not annotated to the above four types of non-coding RNAs, were compared with mature miRNA sequences of the known miRNA in miRBase22 whose screening criteria is no more than two mismatches using BLAST.

Unique reads that were not aligned with the Rfam and miBase databases were compared with the genome using the Mireap online program (http://sourceforge.net/projects/mireap/) to predict the novel miRNAs. The secondary structure maps were drawn using RNAfold. The standardization of expression amount requires inter-sample correction of total Reads, without rather than length correction due to relatively short miRNA length. The gene expression pattern of the sample was comprehensively investigated using count per million (CPM). Differentially expressed miRNAs (DEMs) were analyzed based on the fold difference in expression level (|log2 fold change| > 1) and the significance of the expression difference (*p*-value < 0.05) using DESeq. Finally, target genes for DEMs sequences were predicted using psRobot_tar [52]. The KEGG and GO analysis were performed on all targets to analyze DEMs and target genes function.

### 4.7. Quantitative Real-Time PCR Validation

500 ng of total RNA from each sample was used to synthesize cDNA by PrimerScript RT Mix (Takara, Beijing, China) to validate the mRNA expression level and 1 μg of total RNA from each sample was reverse-transcribed into cDNA using miR-X miRNA First-Strand Synthesis Kit (Takara, Beijing, China) to validate the miRNA expression level. Quantitative real-time PCR (qRT-PCR) analysis was performed using the TB Green Premix Ex TaqII (Takara, Beijing, China) on the CFX Connect Real-Time PCR Detection System (Bio-Rad, Hercules, CA, USA). Relative expression levels of each mRNA and miRNA were determined using 2^−∆∆CT^ method [53]. EF1 [54] and U6 genes were used as the internal reference gene. Three technical replicates were performed for each sample. All primers used in this test were listed in Table S11.

## Figures and Tables

**Table 1:** Changes of FA composition in walnut kernel oil of 80th, 100th, 120th DAF (%).

Types of Fatty Acids			Days after Flowering/Day (MEAN ± SD)		
			80	100	120
Saturated FA		Caprylic acid (C8:0)	0.0077 ± 0.0013 ^a^	0.0071 ± 0.0041 ^a^	0.0065 ± 0.0007 ^a^
		Capric acid (C10:0)	0.0764 ± 0.0140 ^a^	0.0511 ± 0.0079 ^b^	0.0503 ± 0.0090 ^b^
		Myristic acid (C14:0)	0.0400 ± 0.0016 ^a^	0.0264 ± 0.0005 ^b^	0.0224 ± 0.0019 ^c^
		Pentadecanoic acid (C15:0)	0.0455 ± 0.0004 ^a^	0.0221 ± 0.0003 ^b^	0.0172 ± 0.0005 ^c^
		Palmitic acid (C16:0)	7.2106 ± 0.0019 ^a^	5.8410 ± 0.0033 ^c^	5.8890 ± 0.0060 ^b^
		Stearic acid (C18:0)	1.4669 ± 0.0277 ^c^	2.3622 ± 0.0134 ^b^	2.4869 ± 0.0047 ^a^
		Arachidic acid (C20:0)	0.0613 ± 0.0005 ^a^	0.0579 ± 0.0006 ^b^	0.0608 ± 0.0003 ^a^
Unsaturated FA	MUFA	Pentadecenoic acid (C15:1)	0.0078 ± 0.0045 ^a^	0.0053 ± 0.0084 ^a^	0.0083 ± 0.0109 ^a^
		Palmitoleic acid (C16:1)	0.0879 ± 0.0292 ^a^	0.0514 ± 0.0446 ^c^	0.0597 ± 0.0518 ^b^
		Oleic acid (C18:1)	9.7378 ± 0.0306 ^c^	15.3163 ± 0.0435 ^b^	15.7390 ± 0.0526 ^a^
		11-Eicosenoic acid (C20:1)	0.2201 ± 0.0015 ^a^	0.2140 ± 0.0007 ^b^	0.1963 ± 0.0011 ^c^
	PUFA	Linoleic acid (C18:2, n-6)	70.4437 ± 0.0368 ^a^	67.1098 ± 0.0303 ^b^	66.5504 ± 0.0290 ^c^
		γ-Linolenic acid (C18:3, n-6)	0.0363 ± 0.0007 ^a^	0.0350 ± 0.005 ^a^	0.0307 ± 0.0005 ^b^
		α-Linolenic acid (C18:3, n-3)	10.4429 ± 0.0010 ^a^	8.8526 ± 0.0019 ^b^	8.8583 ± 0.0008 ^b^
		11, 14-Eicosadienoic acid (C20:2)	0.0781 ± 0.0123 ^a^	0.0215 ± 0.0021 ^b^	0
		Docosadienoic acid (C22:2)	0.0371 ± 0.0015 ^a^	0.0304 ± 0.0021 ^b^	0.0269 ± 0.0004 ^c^

NOTE: Lowercase (^a^, ^b^, ^c^) in a row indicates significant difference (*p* < 0.05) according to ANOVA with Duncan’s multiple range test.

## Supplementary Materials

Supplementary materials can be found at https://www.mdpi.com/1422-0067/21/23/9093/s1. Raw data of sRNA-seq are available at NCBI, accession number: PRJNA665937.

## Abbreviations

ACC1	acetyl-CoA carboxylase 1
ACOT	acyl-CoA thioesterase
CPM	count per million
DAF	day after flowering
DAG	diacylglycerols
DEG	differentially expressed gene
DEM	differentially expressed miRNA
DGAT	diacylglycerol O-acyltransferase
FA	fatty acid
FabG	3-oxoacyl-[acyl-carrier-protein] reductase 4
FAD	fatty acid desaturase
FATB	palmitoyl-acyl carrier protein thioesterase
GO	Gene Ontology
GPAT	glycerol-3-phosphate acyltransferases
KAR	very long-chain 3-oxoacyl-CoA reductase
KCS	3-ketoacyl-CoA synthase
KEGG	Kyoto Encyclopedia of Genes and Genomes
LACS	long chain acyl-CoA synthetase
LEC1	LEAFY COTYLEDON1
LEC2	LEAFY COTYLEDON2
LPAT	lysophosphatidyl acyltransferase
MUFA	monounsaturated fatty acid
miRNA	microRNA
PAS2	very long-chain (3R)-3-hydroxyacyl-CoA dehydratase 2
PAS2A	very-long-chain (3R)-3-hydroxyacyl-CoA dehydratase PASTICCINO 2A
PAP	phosphatidic acid phosphatase
PC	phosphatidylcholine
PDAT	phospholipid:diacylglycerol acyltransferase
PLA1	phospholipase A I
PLDδ	phospholipase D delta
PUFA	polyunsaturated fatty acid
qRT-PCR	quantitative real-time PCR
rRNA	ribosomal RNA
SAD	stearoyl-[acyl-carrier-protein] 9-desaturase
SLD2	delta 8-fatty-acid desaturase 2
TF	transcription factor
TAG	triacylglycerols

## References

[1] Crews C., Hough P., Godward J., Brereton P., Lees M., Guiet S., Winkelmann W. (2005). Study of the Main Constituents of Some Authentic Walnut Oils. J. Agric. Food Chem..

[2] Guasch-Ferré M., Li J., Hu F.B., Salas-Salvadó J., Tobias D.K. (2018). Effects of walnut consumption on blood lipids and other cardiovascular disease risk factors: An updated meta-analysis and systematic review of controlled trials. Am. J. Clin. Nutr..

[3] Zibaeenezhad M.J., Farhadi P., Attar A., Mosleh A., Amirmoezi F., Azimi A. (2017). Effects of walnut oil on lipid profiles in hyperlipidemic type 2 diabetic patients: A randomized, double-blind, placebo-controlled trial. Nutr. Diabetes.

[4] Shimada T.L., Hara-Nishimura I. (2010). Oil-Body-Membrane proteins and their physiological functions in plants. Biol. Pharm. Bull..

[5] Kagaya Y., Toyoshima R., Okuda R., Usui H., Yamamoto A., Hattori T. (2005). LEAFY COTYLEDON1 controls seed storage protein genes through its regulation of *FUSCA3* and *ABSCISIC ACID INSENSITIVE3*. Plant Cell Physiol..

[6] Mu J.Y., Tan H.L., Zheng Q., Fu F.Y., Liang Y., Zhang J., Yang X.H., Wang T., Chong K., Wang X.J. (2008). LEAFY COTYLEDON1 is a key regulator of fatty acid biosynthesis in *Arabidopsis*. Plant Physiol..

[7] Manan S., Ahmad M.Z., Zhang G., Chen B., Haq B.U., Yang J., Zhao J. (2017). Soybean LEC2 regulates subsets of genes involved in controlling the biosynthesis and catabolism of seed storage substances and seed development. Front. Plant Sci..

[8] Baud S., Guyon V., Kronenberger J., Wuilleme S., Miquel M., Caboche M., Lepiniec L., Rochat C. (2003). Multifunctional acetyl-CoA carboxylase 1 is essential for very long chain fatty acid elongation and embryo development in *Arabidopsis*. Plant J..

[9] Kong Q., Yuan L., Ma W. (2019). WRINKLED1, a “Master Regulator” in transcriptional control of plant oil biosynthesis. Plants.

[10] Kong Y., Chen S., Yang Y., An C. (2013). ABA-insensitive (ABI) 4 and ABI5 synergistically regulate *DGAT1* expression in Arabidopsis seedlings under stress. FEBS Lett..

[11] Yang Y., Yu X., Song L., An C. (2011). ABI4 Activates *DGAT1* expression in *Arabidopsis* seedlings during nitrogen deficiency. Plant Physiol..

[12] Yeap W., Lee F., Shabari Shan D.K., Musa H., Appleton D.R., Kulaveerasingam H. (2017). WRI1-1, ABI5, NF-YA3 and NF-YC2 increase oil biosynthesis in coordination with hormonal signaling during fruit development in oil palm. Plant J..

[13] Hua Y., Zhang C., Shi W., Chen H. (2019). High-throughput sequencing reveals microRNAs and their targets in response to drought stress in wheat (*Triticum aestivum* L.). Biotechnol. Biotechnol. Equip..

[14] Yang M., Lu H., Xue F., Ma L. (2019). Identifying high confidence microRNAs in the developing seeds of *Jatropha curcas*. Sci. Rep..

[15] Jatan R., Tiwari S., Asif M.H., Lata C. (2019). Genome-wide profiling reveals extensive alterations in *Pseudomonas putida*-mediated miRNAs expression during drought stress in chickpea (*Cicer arietinum* L.). Environ. Exp. Bot..

[16] Slocombe S.P., Cornah J., Pinfield-Wells H., Soady K., Zhang Q., Gilday A., Dyer J.M., Graham I.A. (2009). Oil accumulation in leaves directed by modification of fatty acid breakdown and lipid synthesis pathways. Plant Biotechnol. J..

[17] Cao S., Zhu Q., Shen W., Jiao X., Zhao X., Wang M., Liu L., Singh S.P., Liu Q. (2013). Comparative profiling of miRNA expression in developing seeds of high linoleic and high oleic safflower (*Carthamus tinctorius* L.) plants. Front. Plant Sci..

[18] Li J., Ding J., Yu X., Li H., Ruan C. (2019). Identification and expression analysis of critical microRNA-transcription factor regulatory modules related to seed development and oil accumulation in developing *Hippophae rhamnoides* seeds. Ind. Crop. Prod..

[19] Belide S., Petrie J.R., Shrestha P., Singh S.P. (2012). Modification of seed oil composition in *Arabidopsis* by artificial microRNA-mediated gene silencing. Front. Plant Sci..

[20] Li Y., Ma S., Wang Y., Xuan X., Hou L., Sun Q., Yang K. (2012). The dynamics of fat, protein and sugar metabolism during walnut (*Juglans regia* L.) fruit development. Afr. J. Biotechnol..

[21] Chen H., Pan C., Wang B., Xiao Z., Hu Y., Hu G. (2015). Oil Body Observation in Seed Development and Its Analysis in Seed of *Juglans regia* ‘Wen185’ and *J. regia* ‘Xinxin2’ in Period of Seed Maturity. Sci. Agric. Sin..

[22] Zhou L., Quan S., Xu H., Ma L., Niu J. (2018). Identification and Expression of miRNAs Related to Female Flower Induction in Walnut (*Juglans regia* L.). Molecules.

[23] Kim B., Yu H., Park S., Shin J.Y., Oh M., Kim N., Mun J. (2012). Identification and profiling of novel microRNAs in the *Brassica rapa* genome based on small RNA deep sequencing. BMC Plant Biol..

[24] Ye J., Zhang X., Tan J., Xu F., Cheng S., Chen Z., Zhang W., Liao Y. (2020). Global identification of *Ginkgo biloba* microRNAs and insight into their role in metabolism regulatory network of terpene trilactones by high-throughput sequencing and degradome analysis. Ind. Crop. Prod..

[25] Chen C., Zhong Y., Yu F., Xu M. (2020). Deep sequencing identifies miRNAs and their target genes involved in the biosynthesis of terpenoids in *Cinnamomum camphora*. Ind. Crop. Prod..

[26] Pelletier J.M., Kwong R.W., Park S., Le B.H., Baden R., Cagliari A., Hashimotoa M., Munoza M.D., Fischerc R.L., Goldberg R.B. (2017). LEC1 sequentially regulates the transcription of genes involved in diverse developmental processes during seed development. Proc. Natl. Acad. Sci. USA.

[27] Yu J., Zhang Z., Huang S., Han X., Wang X., Pan W., Qin H., Qi H., Yin Z., Qu K. (2019). Analysis of miRNAs targeted storage regulatory genes during soybean seed development based on transcriptome sequencing. Genes.

[28] Wang J., Jian H., Wang T., Wei L., Li J., Li C., Liu L. (2016). Identification of microRNAs actively involved in fatty acid biosynthesis in developing *Brassica napus* seeds using high-throughput sequencing. Front. Plant Sci..

[29] Wang Z., Huang R., Sun Z., Zhang T., Huang J. (2017). Identification and profiling of conserved and novel microRNAs involved in oil and oleic acid production during embryogenesis in *Carya cathayensis* Sarg. Funct. Integr. Genom..

[30] Zheng Y., Chen C., Liang Y., Sun R., Gao L., Liu T., Li D. (2019). Genome-wide association analysis of the lipid and fatty acid metabolism regulatory network in the mesocarp of oil palm (*Elaeis guineensis* Jacq.) based on small noncoding RNA sequencing. Tree Physiol..

[31] Gecgel U., Demirci M., Esendal E., Tasan M. (2007). Fatty Acid Composition of the Oil from Developing Seeds of Different Varieties of Safflower (*Carthamus tinctorius* L.). J. Am. Oil Chem. Soc..

[32] Browse J., McConn M., D James J., Miquel M. (1993). Mutants of Arabidopsis deficient in the synthesis of alpha-linolenate. Biochemical and genetic characterization of the endoplasmic reticulum linoleoyl desaturase. J. Biol. Chem..

[33] Iba K., Gibson S., Nishiuchi T., Fuse T., Nishimura M., Arondel V., Hugly S., Somerville C. (1993). A gene encoding a chloroplast omega-3 fatty acid desaturase complements alterations in fatty acid desaturation and chloroplast copy number of the fad7 mutant of *Arabidopsis thaliana*. J. Biol. Chem..

[34] Zhao S., Zhang X., Su Y., Chen Y., Liu Y., Sun M., Qi G. (2018). Transcriptome analysis reveals dynamic fat accumulation in the walnut kernel. Int. J. Genom..

[35] Lindqvist Y., Huang W., Schneider G., Shanklin J. (1996). Crystal structure of delta9 stearoyl-acyl carrier protein desaturase from castor seed and its relationship to other di-iron proteins. Embo J..

[36] Mattison C.P., Rai R., Settlage R.E., Hinchliffe D.J., Madison C., Bland J.M., Brashear S., Graham C.J., Tarver M.R., Florane C. (2017). RNA-Seq analysis of developing Pecan (*Carya illinoinensis*) embryos reveals parallel expression patterns among allergen and lipid metabolism genes. J. Agric. Food Chem..

[37] Bouvier-Nave P., Benveniste P., Oelkers P., Sturley S.L., Schaller H. (2000). Expression in yeast and tobacco of plant cDNAs encoding acyl CoA:diacylglycerol acyltransferase. Eur. J. Biochem..

[38] Jako C., Kumar A., Wei Y., Zou J., Barton D.L., Giblin E.M., Covello P.S., Taylor D.C. (2001). Seed-specific over-expression of an *Arabidopsis* cDNA encoding a diacylglycerol acyltransferase enhances seed oil content and seed weight. Plant Physiol..

[39] Stahl U., Carlsson A.S., Lenman M., Dahlqvist A., Huang B., Banas W., Banas A., Stymne S. (2004). Cloning and functional characterization of a phospholipid:diacylglycerol acyltransferase from *Arabidopsis*. Plant Physiol..

[40] Pan X., Siloto R.M., Wickramarathna A.D., Mietkiewska E., Weselake R.J. (2013). Identification of a pair of phospholipid:diacylglycerol acyltransferases from developing flax (*Linum usitatissimum* L.) seed catalyzing the selective production of trilinolenin. J. Biol. Chem..

[41] Zhang M., Fan J., Taylor D.C., Ohlrogge J.B. (2010). DGAT1 and PDAT1 acyl transferases have overlapping functions in Arabidopsis triacylglycerol biosynthesis and are essential for normal pollen and seed development. Plant Cell.

[42] Silva G.F.F.E., Silva E.M., Da Silva Azevedo M., Guivin M.A.C., Ramiro D.A., Figueiredo C.R., Carrer H., Peres L.E.P., Nogueira F.T.S. (2014). microRNA156-targeted SPL/SBP box transcription factors regulate tomato ovary and fruit development. Plant J..

[43] Stief A., Altmann S., Hoffmann K., Pant B.D., Scheible W., Bäurle I. (2014). *Arabidopsis* miR156 regulates tolerance to recurring environmental stress through SPL transcription factors. Plant Cell.

[44] Dörmann P., Voelker T.A., Ohlrogge J.B. (2000). Accumulation of palmitate in *Arabidopsis* mediated by the Acyl-Acyl carrier protein Thioesterase FATB1. Plant Physiol..

[45] Chen M., Markham J.E., Cahoon E.B. (2012). Sphingolipid Δ8 unsaturation is important for glucosylceramide biosynthesis and low-temperature performance in *Arabidopsis*. Plant J..

[46] Roesler K., Shintani D., Savage L., Boddupalli S., Ohlrogge J. (1997). Targeting of the *Arabidopsis* homomeric acetyl-coenzyme A carboxylase to plastids of rapeseeds. Plant Physiol..

[47] Somers D.A., Keith R.A., Egli M.A., Marshall L.C., Gengenbach B.G., Gronwald J.W., Wyse D.L. (1993). Expression of the *Acc1* Gene-Encoded Acetyl-Coenzyme A Carboxylase in Developing Maize (Zea mays L.) Kernels. Plant Physiol..

[48] Liu X., Luo X., Luo K., Liu Y., Pan T., Li Z., Duns G.J., He F., Qin Z. (2019). Small RNA sequencing reveals dynamic microRNA expression of important nutrient metabolism during development of *Camellia oleifera* fruit. Int. J. Biol. Sci..

[49] Martinez-Garcia P.J., Crepeau M.W., Puiu D., Gonzalez-Ibeas D., Whalen J., Stevens K.A., Paul R., Butterfield T.S., Britton M.T., Reagan R.L. (2016). The walnut (*Juglans regia*) genome sequence reveals diversity in genes coding for the biosynthesis of non-structural polyphenols. Plant J..

[50] Kim D., Langmead B., Salzberg S.L. (2015). HISAT: A fast spliced aligner with low memory requirements. Nat. Methods.

[51] Wang L., Feng Z., Wang X., Wang X., Zhang X. (2010). DEGseq: An R package for identifying differentially expressed genes from RNA-seq data. Bioinformatics.

[52] Wu H., Ma Y., Chen T., Wang M., Wang X. (2012). PsRobot: A web-based plant small RNA meta-analysis toolbox. Nucleic Acids Res..

[53] Livak K.J., Schmittgen T.D. (2001). Analysis of relative gene expression data using real-time quantitative PCR and the 2−ΔΔCT method. Methods.

[54] Li X., Pan X., Zhang W., Zhang R., Chen J. (2017). Stability evaluation of reference genes for quantitative real-time PCR analysis in walnut (*Juglans* spp.). Plant Physiol. J..

