# Supplementary figures and images for: Integrated Analysis of Seed microRNA and mRNA Transcriptome Reveals Important Functional Genes and microRNA-Targets in the Process of Walnut (Juglans regia) Seed Oil Accumulation
Source: Int J Mol Sci. 2020 Nov 29;21(23):9093. doi: 10.3390/ijms21239093 (PMC7731449; doi:10.3390/ijms21239093)

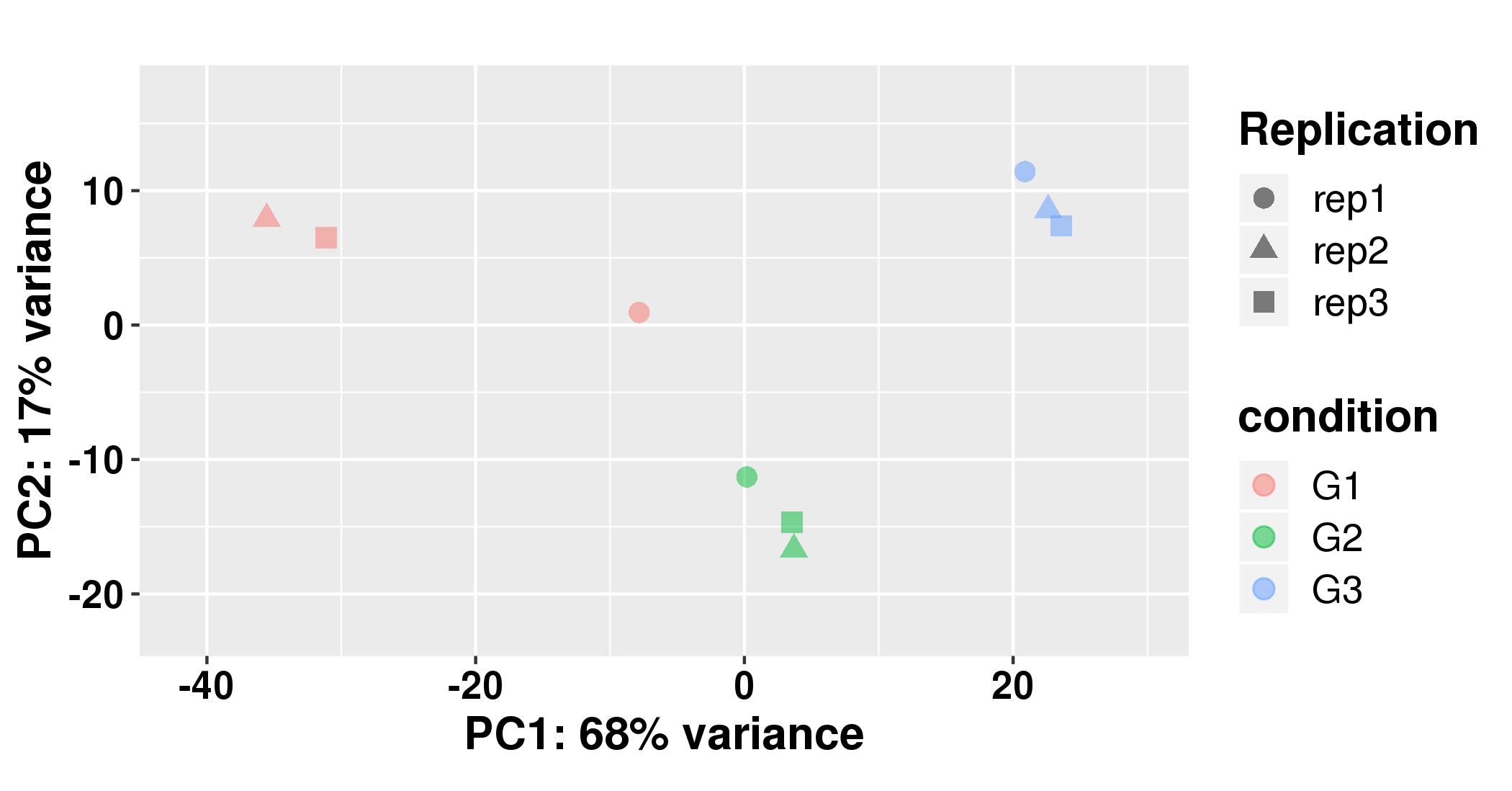

Supplement: Supplementary file 1 [file ijms-21-09093-s001.zip › Figure S2.tif]

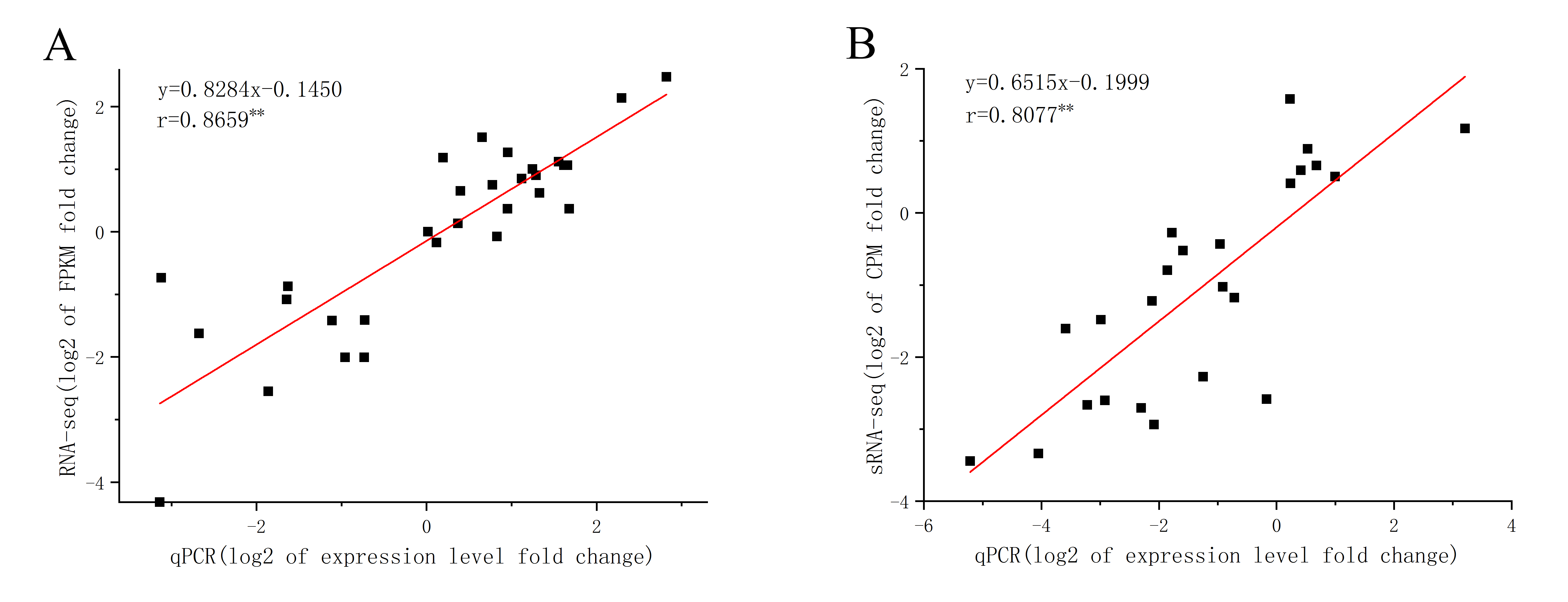

Supplement: Supplementary file 1 [file ijms-21-09093-s001.zip › Figure S3.tif]
